# Process Parameters Optimization Using Taguchi-Based Grey Relational Analysis in Laser-Assisted Machining of Si_3_N_4_

**DOI:** 10.3390/ma14030529

**Published:** 2021-01-22

**Authors:** Yezhuang Pu, Yugang Zhao, Jianbing Meng, Guoyong Zhao, Haiyun Zhang, Qian Liu

**Affiliations:** School of Mechanical Engineering, Shandong University of Technology, Zibo 255049, China; puyezhuang@sdut.edu.cn (Y.P.); jianbingmeng@sdut.edu.cn (J.M.); zgy709@sdut.edu.cn (G.Z.); liuqian@sdut.edu.cn (Q.L.)

**Keywords:** laser-assisted machining, silicon nitride ceramic, surface roughness, work hardening, process parameters

## Abstract

Despite extensive research over the past three decades proving that laser-assisted machining (LAM) is effective for machining ceramic materials, which are affected by many machining parameters, there has been no systematic study of the effects of process parameters on surface quality in LAM ceramic materials. In this paper, the effects and optimization of laser power, spindle speed, feed rate, and cutting depth on surface roughness and work hardening of LAM Si_3_N_4_ were systematically studied, using grey relational analysis coupled with the Taguchi method. The results show that the combination of machining parameters determines the material removal mode at the material removal location, and then affects the surface quality. In ductile material removal mode, both the value of surface roughness and work hardening degree are smaller. Decreased surface roughness and work hardening degree can be obtained with smaller cutting depth and higher laser power.

## 1. Introduction

As an important engineering ceramic, silicon nitride (Si_3_N_4_) ceramic has been widely used in aerospace, national defense and the military industry, modern medicine, and other important fields. This is because of its many excellent properties, such as high strength that can be maintained up to 1200 °C without decreasing, heat shock resistance, wear resistance, and chemical corrosion resistance [1,2]. However, the bonding mode of the covalent bond gives it the characteristics of high hardness and high brittleness. It is easy to produce micro-cracks on the surface and subsurface when Si_3_N_4_ is machined using a conventional cutting method, such as grinding, which greatly reduces the functional performance of the workpiece. The fundamental reason is that the material is removed by way of brittle fracture rather than plastic deformation. Electrical discharge machining (EDM) can machine ceramic materials irrespective of their shape, high wear resistance, corrosion resistance, or toughness [3]. However, the materials machined using EDM must be conductive. Because of the large processing space and the potential to achieve plastic state machining regardless of whether the material is conductive or not, and to achieve excellent surface integrity, laser-assisted machining (LAM) has become the main research focus in recent years. In LAM, before being removed by a cutting tool, the material in the area to be cut is heated to the appropriate temperature via the irradiation of a laser beam, so that the yield strength of the material at this temperature can be reduced to a value below the fracture strength, the material can be softened, and the hardness decreased. This way, the material can be removed by plastic deformation rather than brittle fracture.

Extensive research has been conducted on various ceramics and has proved in the past few decades that LAM is effective for machining brittle and hard materials [4,5,6,7,8,9,10,11,12,13,14,15,16,17,18,19,20]. In the early 1990s, scholars began to study the LAM of engineering ceramics [21]. Lei et al. [4] researched the material removal mechanism of LAM Si_3_N_4_ by using a scanning electron microscope (SEM) to observe the microstructure of chips. When the temperature of the workpiece exceeds the glass transition temperature, under the action of tool load, rod-like Si_3_N_4_ grains in the shear zone slide and rotate along the grain boundary, and the liquid glass phase materials flow to form a new grain boundary. In this state, the chips are separated from the workpiece by intergranular fracture, and the material is removed plastically. Lei et al. [5] investigated the effect of the workpiece temperature on surface roughness. The results showed that workpiece temperature under the operating conditions set in this paper has little effect on surface roughness. The thickness of the influence layer is between 2 and 4 μm. Tian et al. [6] successfully completed LAM of the Si_3_N_4_ workpiece with complex geometry via in-process control of laser power, and studied the surface integrity of the workpiece. Shen et al. [7] established the transient thermal model for laser assisted milling (LAMill) of Si_3_N_4_ using the finite element analysis method. The results of verified experiments show that laser power is one of the key parameters for the success of LAMill. Lee et al. [8] studied the changes of microstructural and machining characteristics of Si_3_N_4_ in LAM. With the increase of temperature, Si_3_N_4_ decomposes into SiO_2_ and N_2_, which erupts outwards, forming a porous structure on the surface. Pu et al. [9] studied the relationship between the laser power and surface topography parameters with a single-factor experiment. The results show that the values of *Sa* and *Sq* are smaller in ductile material removal mode.

From the literature study, it can be seen that the research on surface integrity, cutting force, and tool life proves the effectiveness of LAM in machining ceramic materials. However, there has been no systematic study of the effects of process parameters on surface integrity in LAM ceramic materials. Surface integrity, which is reliant on many parameters, greatly affects the functional performance of parts. In LAM, the multi-field coupling formed by laser, shear stress, large strain rate, and shock wave produces a joint effect on the material removed region, and further improves the process complexity. Therefore, these parameters include spindle speed, feed rate, cutting depth, laser power, preheating time, diameter of the laser facula, axial distance between laser spot and tool, circumferential laser-tool angle, and so on. As long as one parameter does not match other parameters, the best surface integrity cannot be obtained. The simple way to improve the surface integrity is to optimize the parameter combination of spindle speed, feed speed, cutting depth, and laser power when other parameters are fixed. This is because compared with other parameters, these parameters can be adjusted in the process of machining, which is convenient for realizing automation and intelligent processing.

Grey system theory takes an uncertain system as a research object, seeking the rules from the known information and mining the unknown information [22]. Grey relational analysis (GRA) coupled with the Taguchi method can achieve multi-objective optimization, which overcomes the disadvantage that the Taguchi method can only carry out single-objective optimization. Grey-based Taguchi methods have been used in many machining and manufacturing fields for carrying out multi-criteria optimization [23,24,25,26,27,28,29].

In this study, the effects and optimization of process parameters such as laser power, spindle speed, feed rate, and cutting depth on surface roughness and work hardening are analyzed. The relationship among process parameters, surface roughness, work hardening, and material removal mode was also studied for a comprehensive analysis of the machining mechanism.

## 2. LAM Experiment

### 2.1. Experimental System

**Figure 1:**
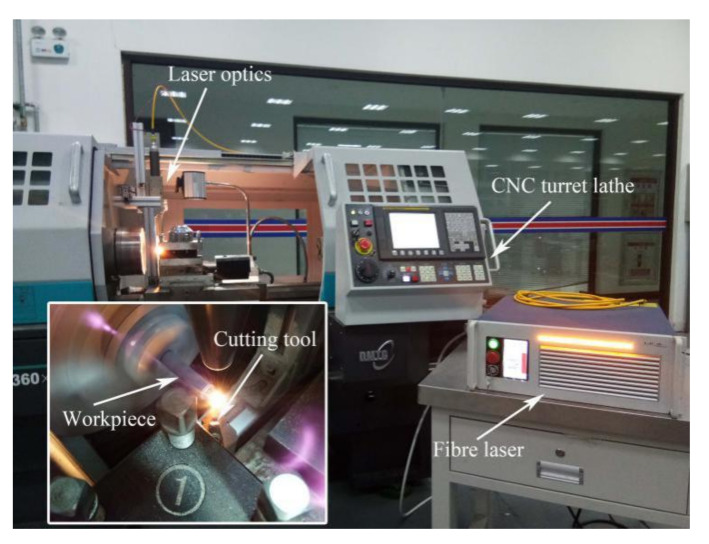
Experimental system for laser-assisted machining (LAM) of Si_3_N_4_.

The experimental system for LAM Si_3_N_4_ is illustrated in Figure 1. A CNC turret lathe (Dalian Machine Tools Group, Dalian, China) was used to perform the machining test using CBN-tipped tool inserts with MCLNR2020K12 tool holders (Halnn Superhard, Zhengzhou, China).

A high-power laser beam generated from an ytterbium fiber laser with a maximum average power of 250 W in continuous wave mode and a wavelength of 1070 nm was focused on the workpiece surface through laser optics. The laser optics were mounted on a fixture attached to the apron, and could synchronously move with the cutting tool along the axial direction.

### 2.2. Experimental Material

The dimensions of the gas-pressure sintered cylindrical Si_3_N_4_ workpiece used for the experiment were *Ф* 10 × 100. A three-jaw chuck was used to hold the workpiece. The material’s properties are listed in Table 1, and these values were measured by the material’s manufacturer.

### 2.3. Experimental Matrix and Operating Parameters

**Figure 2:**
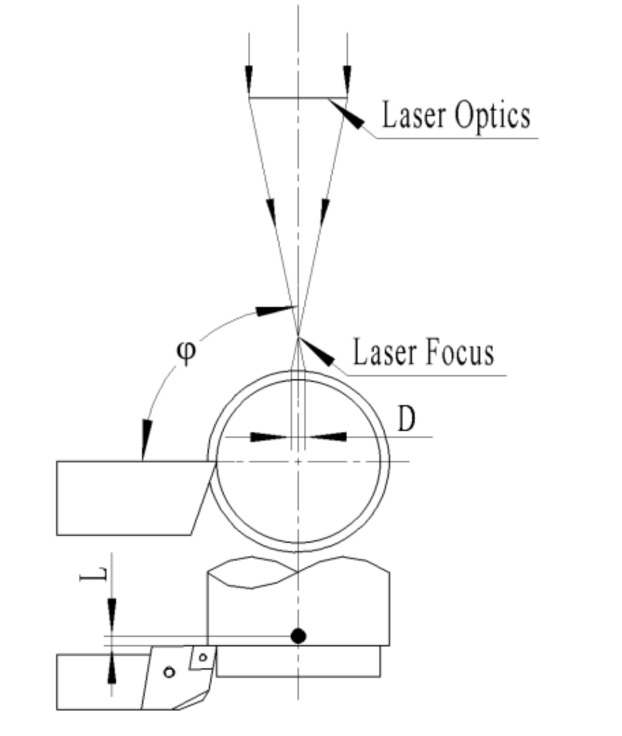
Schematic diagram of LAM.

The main machining conditions are given in Table 2, where laser power (*P*, continuous wave mode), spindle speed (*S*), feed rate (*f*), and cutting depth (*a_p_*) were studied for their effects on surface quality based on GRA coupled with the Taguchi method. The experimental scheme and results are shown in Table 3. There are many other parameters affecting the LAM characteristics besides the above four factors. In this study, the length of the cut (*l*), preheating time (*t*), diameter of the laser facula (*D*), laser-tool distance (*L*, axial distance between laser spot and tool edge), and circumferential laser-tool angle (*φ*) were fixed as 15 mm, 8 s, 1 mm, 0.5 mm, and 90°, respectively. Figure 2 is the schematic diagram of the relative positions of the laser optics, the cutting point, and the workpiece. The values of parameters were determined by the simulation of temperature field using ANSYS software and single-factor experiment based on the previous research. To ensure the correct results, each group of parameters was repeated twice.

The surface roughness (*Ra*) of each workpiece was measured seven times in seven randomly selected positions using a portable surface profiler. The evaluation lengths and cut-off lengths were set to 4 mm and 0.8 mm, respectively, with no electronic filter. The surface roughness value is the average value of the remaining value after removing a maximum value and a minimum value.

Micro-hardness was measured with an FM-800 micro-hardness tester (TIME, Shanghai, China) with 9.8 N and 15 s dwell time. For each workpiece, seven measurements were conducted and a maximum value and a minimum value were removed. The remaining values were then averaged to obtain the micro-hardness value. Work hardening degree *N_H_* (Equation (1)) is defined as follows:

(1) $$N_{H}=\frac{HV}{HV_{0}}\times 100\%$$

where *HV* is the micro-hardness of the machined surface, and *HV*_0_ is the micro-hardness of the as-received workpiece, which is 1622 HV in this paper.

## 3. Single Objective Optimization

### 3.1. Analysis of the S/N Ratio

The *S/N* (Equation (2)) ratio is usually used as a quantitative tool. The higher the *S/N* ratio, the better. As the output response, surface roughness needs to be at a minimum; therefore, the *S/N* ratio is defined as follows:

(2) $$S/N=-10\log\left[\frac{1}{n}(y_{1}^{2}+y_{2}^{2}+\cdots +y_{n}^{2})\right]$$

where *S/N* represents the response values (unit: dB) and *y*^1^, *y*^2^, …, *y^n^* are the observed values of the output for a trial condition repeated *n* times.

Moderate work hardening can improve the wear resistance of the workpiece. However, work hardening can reduce the plasticity and toughness, and too much work hardening can cause microcracks on the machined surface, which will reduce the fatigue life of the parts. This moderate value depends on the specific application. In this study, work hardening is also considered to be at a minimum.

**Figure 3:**
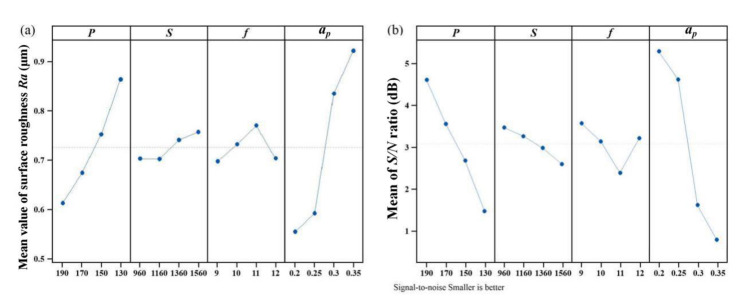
Main effect plots: (**a**) the effects of input factors on surface roughness and (**b**) the mean S/N ratios corresponding to surface roughness.

**Figure 4:**
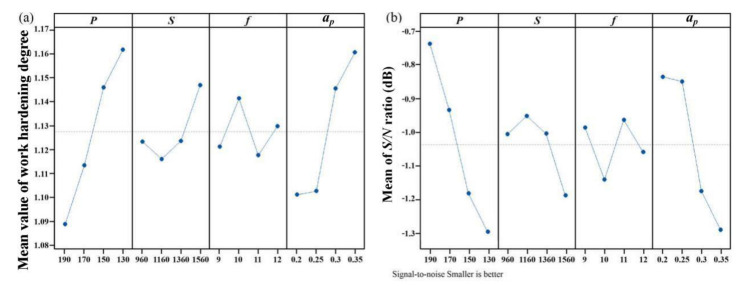
Main effect plots: (**a**) the effects of input factors on work hardening and (**b**) the mean S/N ratios corresponding to work hardening.

Figure 3a and Figure 4a show the mean value of surface roughness and work hardening degree, respectively. The most important factor affecting surface roughness is cutting depth, followed by laser power, as shown in Figure 3a. The most important factor affecting work hardening is laser power, followed by cutting depth, as shown in Figure 4a. With increasing cutting depth, the softening degree of the cutting area is reduced, and the surface roughness and work hardening degree increase. With increasing laser power, the softening degree of the cutting area increases, and the surface roughness and work hardening degree decrease. The higher the spindle speed is, the shorter the laser irradiation time is, the lower the softening degree is, and the greater the surface roughness is. Figure 3b shows that cutting depth and laser power have the greatest effect on surface roughness. Figure 4b shows that laser power and cutting depth have the greatest effect on work hardening degree. The optimal parameters and their levels for surface roughness are A1B1C1D1, and the optimal parameters and their levels for work hardening degree are A1B2C3D1, as seen in the S/N ratio analyses in Figure 3b and Figure 4b.

### 3.2. Analysis of Variance

In order to study the significance of the input parameters on surface quality, analysis of variance (ANOVA) was applied. From Table 4, it is evident that cutting depth and laser power are the main factors affecting surface roughness at a 95% confidence level, because their P-values are less than 0.05, and their percentage contributions are 69.84% and 25.20%, respectively. From Table 5, it is evident that laser power and cutting depth are the main factors affecting work hardening at a 95% confidence level, because their P-values are less than 0.05, and their percentage contributions are 45.16% and 38.51%, respectively. Spindle speed and feed rate in the parameters range do not cause great changes in surface roughness or work hardening.

## 4. Multi-Objective Optimization

### 4.1. Normalizing the Response Data

The dimensions of different responses are different, so it is necessary to convert the original data into a common dimensionless quantity. The formula for the normalization method is (Equation (3)) [29]:

(3) $$y_{i}(k)=\frac{\max x_{i}(k)-x_{i}(k)}{\max x_{i}(k)-\min x_{i}(k)}$$

where *y_i_(k)* is the sequence after data normalizing, *x_i_(k)* is the original response sequence, *max x_i_(k)* is the maximum value of *x_i_(k)*, and *min x_i_(k)* is the minimum value of *x_i_(k)*.

### 4.2. Calculating the Deviation Sequence

The calculation formula is as follows (Equation (4)) [29]:

(4) $$\Delta_{i}(k)=\left|y_{i}^{0}(k)-y_{i}^{}(k)\right|$$

where $\Delta_{i}(k)$ is the deviation sequence, and $y_{i}^{0}(k)$ is the reference sequence. In this study, the reference values of surface roughness and work hardening degree are 1.

### 4.3. Calculating the Grey Relational Coefficient

The calculation formula is as follows (Equation (5)) [29]:

(5) $$\gamma_{ik}=\frac{m+\zeta \cdot M}{\Delta_{i}k+\zeta \cdot M}$$

where $M=\underset{i}{\max}\;\underset{k}{\max}\;\Delta_{i}(k)$, and $m=\underset{i}{\min}\;\underset{k}{\min}\;\Delta_{i}(k)$. The distinguishing coefficient ζ is defined in the range of 0<ζ<1, and ζ is generally taken as 0.5.

### 4.4. Calculating the Grey Relational Grade (GRG)

The calculation formula is as follows (Equations (6) and (7)) [29]:

(6) $$\gamma_{k}=\sum_{i=1}^{n}\omega_{i}\cdot \gamma_{ik}$$

(7) $$\sum_{i=1}^{n}\omega_{i}=1$$

where *ω_i_* is the weight of the *i*th input variable. The calculated grey relational coefficient and GRG values are listed in Table 6. The multi-response optimization problem has been transformed into a single equivalent objective function optimization problem, using a combination of the Taguchi approach and GRA. When the value of GRG is higher, the corresponding factor combination is said to be close to the optimal.

### 4.5. Taguchi-Based GRA

GRA coupled with the Taguchi method was used to determine the optimal parameter settings. Table 7 shows the optimal parameters for better surface finish, and the effect of each parameter on response variables.

From Table 7, the following are noticeable:

The grey relational order of the effect of laser power on the responses is as follows:

$$\gamma_{190W}>\gamma_{170W}>\gamma_{150W}>\gamma_{130W}.$$

The grey relational order of the effect of spindle speed on the responses is as follows:

$$\gamma_{960rev/\min}>\gamma_{1160rev/\min}>\gamma_{1360rev/\min}>\gamma_{1560rev/\min}.$$

The grey relational order of the effect of feed rate on the responses is as follows:

$$\gamma_{9mm/\min}>\gamma_{10mm/\min}>\gamma_{12mm/\min}>\gamma_{11mm/\min}.$$

The grey relational order of the effect of cutting depth on the responses is as follows:

$$\gamma_{0.2mm}>\gamma_{0.25mm}>\gamma_{0.3mm}>\gamma_{0.35mm}.$$

Consequently, the optimum parameter setting is A1B1C1D1.

## 5. Effect of Material Removal Mode on Surface Quality

Under different combinations of process parameters, the material removal mode determined by the softening degree at the material removal location is different, thereby affecting the surface quality. A large volume of research results of LAM of engineering ceramics shows that only when the material reaches the appropriate softening degree under the irradiation of the laser can the plastic removal be realized and excellent surface integrity obtained [4,5,6,7,8,9]. Overheating will cause burning on the machined surface, or will cause the material to still be removed in the way of brittle fracture due to insufficient softening.

**Figure 5:**
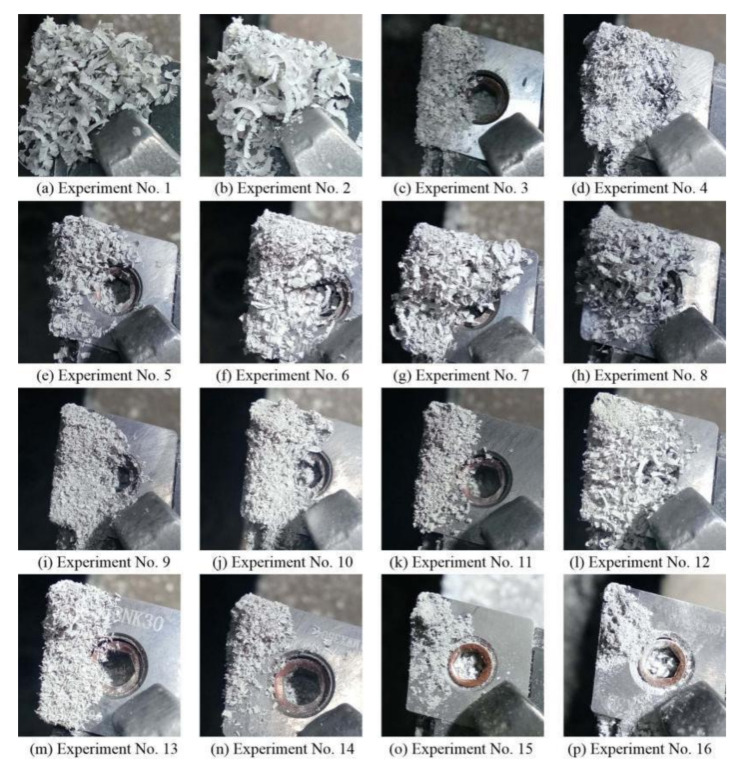
Macrographs of chips for LAM Si_3_N_4_. (**a**)–(**p**) Experiment No. 1–16.

Macrographs of the chips are shown in Figure 5. Continuous band-shaped chips can be seen in experiments 1–3, 5–8, 11, and 12. Continuous band-shaped chips show that the material was removed plastically, which is also the performance of good process parameters. According to Lei et al.’s research [4], larger chips can be produced because of delayed break formation at the higher temperature. In experiments 4, 13, and 14, the band-shaped chips became needle-like, which shows that the material removal mode had reached the critical state of plastic removal. Particle-like chips indicate that the material was removed by brittle fracture in experiments 9, 10, 15, and 16.

**Figure 6:**
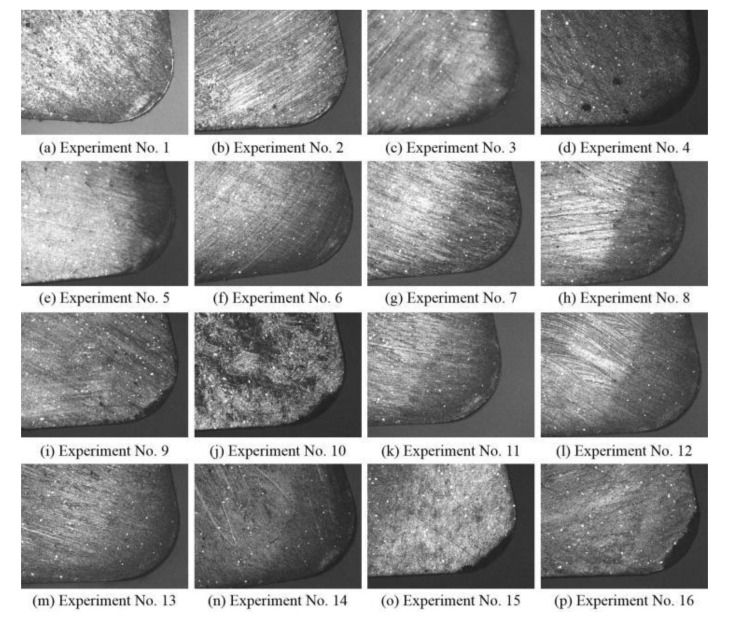
Micrographs of the CBN inserts. (**a**)–(**p**) Experiment No. 1–16.

The tool wear of each experiment, observed afterward using an optical microscope at a magnification of 50, is shown in Figure 6. It can be seen clearly that the tool wear form of experiments 9, 10, 15, and 16 was cutting edge tipping, and there are friction marks on the rake face in the rest. When the continuous band-shaped chips flow out along the rake face, adhesion and friction will occur between the chip and the tool, resulting in the wear of the rake face. With the decrease of the temperature in the cutting zone, the continuous band-shaped chips became particle-like chips. The contact time between the particle-like chips and the cutting tool was very short, and the contact area was concentrated in the narrow area near the cutting edge, resulting in the tipping of the cutting edge.

**Figure 7:**
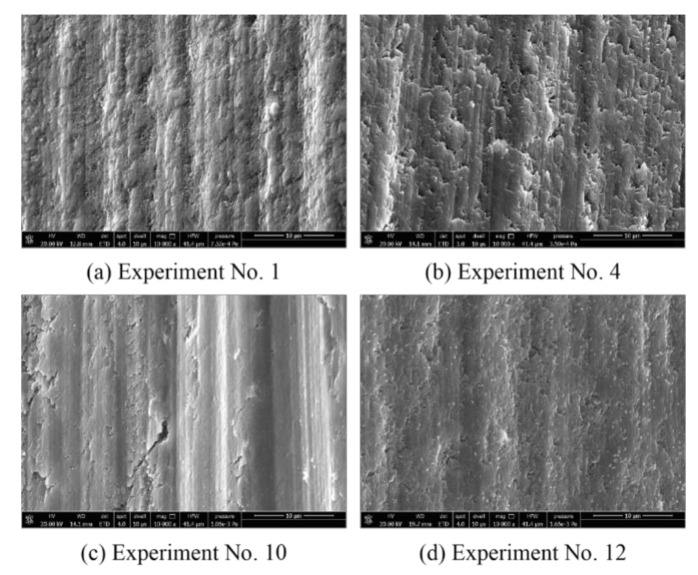
The SEM images of the machined surface. (**a**)–(**d**) Experiment No. 1, 4, 10 and 12.

The SEM images of the machined surface are shown in Figure 7. Cracks and pits can be seen on the machined surface of experiment 10. The machined surface of experiments 1, 4, and 12 are extremely smooth, with no cracks or pits.

In summary, in experiments 1–8 and 11–14, the material plastic removal was dominant. In experiments 9, 10, 15, and 16, brittle fracture became the main material removal method. The surface quality obtained in the plastic machining state was better than that obtained when the material was removed in the way of brittle fracture. Therefore, the material removal mode, which is determined by the combination of process parameters, is the most important factor affecting the surface quality.

## 6. Conclusions

The effects of process parameters such as laser power, spindle speed, feed rate, and cutting depth on surface roughness and work hardening during the LAM of Si_3_N_4_ were studied using GRA coupled with the Taguchi method, and the following conclusions were obtained:

1. From the main effect plots and *S/N* ratios, it is evident that the most influential factor for surface roughness is cutting depth, followed by laser power. The most influential factor for work hardening is laser power, followed by cutting depth. Decreased surface roughness and work hardening degree can be obtained with smaller cutting depth and higher laser power.
2. From the ANOVA analysis, it is evident that the influence of process parameters on surface quality is varied. The contribution rates of cutting depth and laser power for surface roughness are 69.84% and 25.20%, respectively, and the contribution rates of spindle speed and feed rate are less than 3%. The contribution rates of laser power and cutting depth for work hardening are 45.16% and 38.51%, respectively, and the contribution rates of spindle speed and feed rate are less than 8%.
3. The optimal condition for attaining decreased surface roughness and work hardening degree based on the grey-Taguchi method is A1B1C1D1, which is 190 W laser power, 960 rev/min spindle speed, 9 mm/min feed rate, and 0.2 mm cutting depth.
4. The combination of process parameters determines the material removal mode at the material removal location, and then affects the surface roughness and work hardening. In ductile material removal mode, the values of surface roughness and work hardening degree are lower.

## Figures and Tables

**Table 1:** Properties of Si_3_N_4_ workpieces measured by the manufacturer.

Content	Values
Density (g/cm^3^)	3.2 ± 0.05
Hardness (HV)	≥1420
Fracture toughness (MPa·m^1/2^)	6.0–7.0
Flexural strength (MPa)	700–800
Elastic modulus (GPa)	310
Thermal expansion (room temperature ~500 °C) 10^−6^/°C	3.0–3.2
Breakdown voltage (KV)	>10
Compressive strength (MPa)	≥1500
Thermal conductivity (W/mK)	15–20

**Table 2:** The main machining conditions.

Factors	Parameters	Levels			
		1	2	3	4
a	Laser power (W)	190	170	150	130
b	Spindle speed (rev/min)	960	1160	1360	1560
c	Feed rate (mm/min)	9	10	11	12
d	Cutting depth (mm)	0.2	0.25	0.3	0.35

**Table 3:** The experiment parameters and results.

No.	Factor a	Factor b	Factor c	Factor d	Surface Roughness		Work Hardening Degree	
					Mean Value (μm)	S/N Ratio	Mean Value (%)	S/N Ratio
1	1	1	1	1	0.413	7.689	106.2	−0.520
2	1	2	2	2	0.478	6.418	107.2	−0.605
3	1	3	3	3	0.757	2.419	108.1	−0.675
4	1	4	4	4	0.807	1.865	114.1	−1.147
5	2	1	2	3	0.755	2.439	113.9	−1.128
6	2	2	1	4	0.794	2.002	111.7	−0.957
7	2	3	4	1	0.511	5.840	109.1	−0.758
8	2	4	3	2	0.637	3.914	110.8	−0.890
9	3	1	3	4	0.984	0.143	117.1	−1.369
10	3	2	4	3	0.837	1.548	116.5	−1.324
11	3	3	1	2	0.595	4.506	110.9	−0.895
12	3	4	2	1	0.594	4.522	114.1	−1.142
13	4	1	4	2	0.661	3.598	112.3	−1.005
14	4	2	3	1	0.703	3.066	111.2	−0.919
15	4	3	2	4	1.101	−0.840	121.5	−1.688
16	4	4	1	3	0.990	0.084	119.9	−1.573

**Table 4:** Results of the ANOVA for surface roughness.

Variation in Source	Degree of Freedom (DF)	Sum-of-Squares (SS)	Mean-of-Squares (MS)	F-Value	*p*-Value	Contribution
Laser power	3	0.1401	0.0467	25.459	0.012	25.20%
Spindle speed	3	0.0090	0.0030	1.6439	0.347	1.63%
Feed rate	3	0.0130	0.0043	2.365	0.249	2.34%
Cutting depth	3	0.3882	0.1294	70.551	0.003	69.84%
Error	3	0.0055	0.0018	–	–	0.99%
Total	15	0.5559	–	–	–	–

**Table 5:** Results of the ANOVA for work hardening.

Variation in Source	Degree of Freedom (DF)	Sum-of-Squares (SS)	Mean-of-Squares (MS)	F-Value	*p*-Value	Contribution
Laser power	3	0.0128	0.00428	11.260	0.039	45.16%
Spindle speed	3	0.0022	0.00072	1.895	0.307	7.60%
Feed rate	3	0.0013	0.00045	1.176	0.449	4.72%
Cutting depth	3	0.0110	0.00365	9.601	0.048	38.51%
Error	3	0.0011	0.00038	–	–	4.01%
Total	15	0.0284	–	–	–	–

**Table 6:** Grey relational coefficient and GRG.

Experiment No.	Grey Relational Coefficient		GRG	Rank
	Surface Roughness	Work Hardening Degree		
1	1	1	1	1
2	0.841	0.884	0.858	2
3	0.500	0.801	0.620	5
4	0.466	0.492	0.476	12
5	0.501	0.498	0.500	11
6	0.474	0.582	0.517	10
7	0.778	0.725	0.757	3
8	0.606	0.624	0.613	6
9	0.376	0.412	0.391	14
10	0.448	0.426	0.439	13
11	0.654	0.619	0.640	4
12	0.655	0.492	0.590	7
13	0.581	0.556	0.571	8
14	0.543	0.605	0.567	9
15	0.333	0.333	0.333	16
16	0.374	0.358	0.367	15

**Table 7:** Response table for GRG.

Process Parameter	Average GRG			
	Level 1	Level 2	Level 3	Level 4
Laser power	0.739 *	0.597	0.515	0.460
Spindle speed	0.615 *	0.596	0.588	0.512
Feed rate	0.631 *	0.570	0.548	0.561
Cutting depth	0.729 *	0.671	0.482	0.429

‘*’ means the best choice.

## Data Availability

Data sharing is not applicable to this manuscript.
